# Breakage of CRISPR/Cas9-Induced Chromosome Bridges in Mitotic Cells

**DOI:** 10.3389/fcell.2021.745195

**Published:** 2021-09-28

**Authors:** Marina Rodriguez-Muñoz, Martina Serrat, David Soler, Anna Genescà, Teresa Anglada

**Affiliations:** Department of Cell Biology, Physiology and Immunology, Universitat Autònoma de Barcelona, Bellaterra, Spain

**Keywords:** DNA bridges, genomic instability, chromosome damage, mitosis, DNA repair

## Abstract

Chromosomal instability, the most frequent form of plasticity in cancer cells, often proceeds through the formation of chromosome bridges. Despite the importance of these bridges in tumor initiation and progression, debate remains over how and when they are resolved. In this study, we investigated the behavior and properties of chromosome bridges to gain insight into the potential mechanisms underlying bridge-induced genome instability. We report that bridges may break during mitosis or may remain unbroken until the next interphase. During mitosis, we frequently observed discontinuities in the bridging chromatin, and our results strongly suggest that a substantial fraction of chromosome bridges are broken during this stage of the cell cycle. This notion is supported by the observation that the chromatin flanking mitotic bridge discontinuities is often decorated with the phosphorylated form of the histone H2AX, a marker of DNA breaks, and by MDC1, an early mediator of the cell response to DNA breaks. Also, free 3′OH DNA ends were detected in more than half of the bridges during the final stages of cell division. However, even if detected, the DNA ends of broken bridges are not repaired in mitosis. To investigate whether mitotic bridge breakage depends on mechanical stress, we used experimental models in which chromosome bridges with defined geometry are formed. Although there was no association between spindle pole separation or the distance among non-bridge kinetochores and bridge breakage, we found a direct correlation between the distance between bridge kinetochores and bridge breakage. Altogether, we conclude that the discontinuities observed in bridges during mitosis frequently reflect a real breakage of the chromatin and that the mechanisms responsible for chromosome bridge breakage during mitosis may depend on the separation between the bridge kinetochores. Considering that previous studies identified mechanical stress or biochemical digestion as possible causes of bridge breakage in interphase cells, a multifactorial model emerges for the breakage of chromosome bridges that, according to our results, can occur at different stages of the cell cycle and can obey different mechanisms.

## Introduction

An important characteristic of tumors is their ability to adapt. In the face of changes, be they endogenous or exogenous, the neoplastic cell undergoes alterations to generate a variety of phenotypes, some of which give the cell the ability to survive. An important difficulty in combating the adaptive capacity of neoplastic cells is that there is no single mechanism responsible for genome instability, but there are at least four different mechanisms: excessive erosion of telomeric sequences, accelerated DNA replication, impaired DNA repair, and defects in chromosome segregation during mitosis. However, although intrinsically different, the four mechanisms share, to a greater or lesser extent, a common intermediary: the chromosome bridge. This abnormal structure is frequently observed in tumor samples; it occurs 10 times more frequently in neoplasms and premalignant lesions than in healthy tissue (Rudolph et al., 2001; Gisselsson, 2003). Chromosome bridges associate with genomic instability as they can cause the regression of the cleavage furrow and lead to the formation of tetraploid cells in which supernumerary centrosomes hinder the correct segregation of chromosomes (Ganem et al., 2009; Pampalona et al., 2012). Alternatively, bridges can break and lead to DNA damage restricted to the chromosome involved in the bridge, a process known as chromothripsis (Maciejowski et al., 2015, 2020). Despite its close association with genome instability and its importance in tumor initiation and progression, the mechanisms that determine how and when bridges are resolved are unclear.

According to the Breakage-Fusion-Bridge (BFB) model (McClintock, 1939), chromosome bridges can break and initiate a cycle capable of self-feeding when a broken DNA end fuses with another broken end and forms a new unstable chromosome structure. In this way, the BFB cycle can instigate a profound reorganization of the genome. Based on time-lapse observations of living cells, classical studies placed the breakage of chromatin bridges during the last stages of cell division (Hoffelder et al., 2004; Shimizu et al., 2005). However, studies aimed at determining the causes of bridge rupture during mitosis reached controversial conclusions. A rapid shrinking of the chromatin fiber compatible with tangling of the severed chromatin prior to nuclear membrane reformation and completion of cytokinesis was reported by Shimizu et al. (2005). Whereas these observations indicate that mechanical tension would be responsible for severing anaphase bridges, other studies suggest that constricting forces generated by the cleavage furrow break chromosomal DNA (Janssen et al., 2011).

More recent studies argue that the often-visible discontinuities in bridging chromatin during the last stages of mitosis do not correspond to actual breaks (Maciejowski et al., 2015; Umbreit et al., 2020). Unlike the classical models, Maciejowski et al. (2015) and Umbreit et al. (2020) state that chromosome bridges cannot be resolved in anaphase and, therefore, inevitably lead to the presence of chromatin in the cleavage plane. According to these authors, stabilized nucleoplasmic bridges persist in daughter cells for a considerable period of time as the presence of chromatin in the cleavage plane inhibits abscission, the final stage of cytokinesis (Steigemann et al., 2009). From there, destabilization of the bridge due to mechanical stress (Umbreit et al., 2020) or enzymatic digestion after loss of nuclear envelope function (Maciejowski et al., 2015, 2020) will eventually lead to bridge resolution in the interphase. In summary, different models have been proposed to explain how and when chromosome bridges are resolved, and it is unclear at present whether they can break during mitosis or, alternatively, remain invariably unbroken until the end of mitosis to break in the next interphase. It is important to distinguish between these two possibilities, as the mechanisms that determine the breakage of bridges may be different in the different stages of the cell cycle.

By using different methods for the detection of DNA breakage, we found that the discontinuities frequently observed in bridging chromatin at the end of mitosis mostly correspond to real breaks. Whereas some bridges remain unbroken and form nucleoplasmatic bridges, by employing experimental models to produce bridges with defined characteristics, we demonstrate that others resolve during mitosis, and their breakage is associated with the distance between the kinetochores involved in the bridge.

## Materials and Methods

### Cell Culture

HEK 293T and 293T Phoenix cells were cultured in MEM, and U2OS cells were propagated in 1:1 DMEM:Ham’s F10 medium. All media were supplemented with 10% Fetal Bovine Serum (FBS). MCF10A were grown in DMEM:F12 with 5% horse serum, 100 ng/mL cholera toxin, 10 μg/mL insulin, 20 ng/ml epidermal growth factor, and 0.5 μg/mL hydrocortisone. RPE1 Cas9 Tet-ON cells were obtained from Iain Cheeseman’s Laboratory and cultured in DMEM:F12 with 10% tetracycline-free FBS (FBS-TET-12A, Labclinics, Barcelona, Spain). U2OS LacO/LacI-GFP-CENPT were a kind gift from Iain Cheeseman’s Laboratory (Gascoigne et al., 2011) and were cultured in 10 mM IPTG (Isopropyl β-D-1-thiogalactopyranoside, I6758-1G, Merck, Kenilworth, NJ, United States) and 200 μg/mL Hygromycin B (H3274, Merck). U2OS GFP-MDC1 were obtained from the Laboratory of Manuel Stucki and were grown in 250 μg/mL of geneticin (11811023, Thermo Fisher Scientific, Waltham, MA, United States). Penicillin/streptomycin (100 U/mL) was added to the media, and cell cultures were maintained in a humidified incubator at 37°C and 5% CO_2_.

### Plasmid Construction

A retroviral plasmid was obtained by cloning the GFP-Lap2β sequence (a gift from Ulrike Kutay’s Laboratory) into the pLNCX2-mCherry-CHMP4B plasmid (a gift from Sanford Simon; Addgene plasmid #116923) between the *Age*I and *Not*I restriction sites, thus removing the mCherry-CHMP4B gene. Guide RNA for the subtelomeric region of chromosome 2 (5′-ATATTAAGGGCTCCCCGTCG-3′) was designed using the CCTop - CRISPR/Cas9 target online predictor platform (Stemmer et al., 2015) and was cloned into lenti-sgRNA blast (gift from Brett Stringer, Addgene #104993) between the *Bsm*BI restriction sites. The cloning protocol for lenti-sgRNA blast is described in addgene website^1^. All plasmids were verified by Sanger sequencing. After sequencing, plasmids were amplified by transformation into Stbl3 (C737303, Invitrogen, Waltham, MA, United States) or DH5α competent cells (18258012, Thermo Fisher Scientific) and purified using the NucleoBond PC kit (740573.100, Macherey-Nagel, Bethlehem, PA, United States) following the manufacturer’s instructions. The DNA concentration was measured with a NanoDrop 2000 spectrometer (Thermo Fisher Scientific).

### Viral Particle Production and Transduction

Lentivirus or retrovirus particles were produced in HEK 293T or 293T Phoenix, respectively. Cells were transfected at 70% confluency with 1 μg/mL of total DNA plasmid following the protocol from Trono’s Laboratory website^2^. For lentivirus particle production, the DNA concentration of all plasmids was adjusted to a ratio 4:3:1 (transfer plasmid:psPAX2:pMD2.G; Supplementary Table 1). The supernatant was harvested 48 h later, concentrated through centrifugal filters (UFC910008, Merck), and stored at −80°C.

Cells were transduced with viral particles (Supplementary Table 1) overnight using medium with 4 μg/mL polybrene. For retroviral transduction, the procedure was repeated three times. Cells with the antibiotic-resistance cassette were selected, with 2.5 μg/mL blasticidin (15205, Merck) or 250 μg/mL geneticin (11811023, Thermo Fisher Scientific) for 2 weeks or 2 μg/mL puromycin (P8833, Merck) for 1 week. Transduced fluorescent cells were enriched by fluorescent activated cell sorting (FACS) by using a BD FACSJazz (BD Biosciences, Franklin Lakes, NJ, United States) equipped with a 488 nm laser and two detectors (530/40 and 692/40 nm).

### Methods to Generate Chromosome Bridges

Chromosome bridges were generated using different methodologies: (1) To generate bridges with irradiation, cells were exposed to 2.5 Gy using an IBL-437C R-137 Cs irradiator (dose rate of 5.10 Gy/min) and incubated at 37°C and 5% CO_2_ for 24 h prior to fixation. (2) To generate bridges using an inducible ectopic kinetochore function, IPTG was removed from the media to allow the interaction between LacO and LacI-GFP-CENPT (after seven washes with PBS), and the cells were fixed 24 h later. (3) To generate bridges with the CRISPR/Cas9 based model in cells expressing sgRNA targeting subtelomere regions, Cas9 was expressed by adding 1 μg/ml doxycycline (631311, Clontech, Mountain View, CA, United States) to the medium for 15 h, and a washout of 24 h was performed before the cells were fixed. When indicated, cells were synchronized in G2 phase with 9 μM RO3306 (SML0569, Sigma-Aldrich, Saint Louis, MO, United States) for 18 h and were released for 43–120 min.

### BrdU Incorporation

For the BrdU pulse, cells were seeded in 35-mm-diameter petri dishes and after 24 h, 10 μM 5-bromo-2′-deoxyuridine (BrdU) was added to the culture for 8 h and then substituted with 10 μM BrdU for an additional 16 h.

### Immunofluorescence

Cells were fixed with 4% paraformaldehyde (PFA) for 15 min, permeabilized with 1x PBS/0.5% Triton-X-100 solution for 20 min, and blocked with 1x PBS/0.5% BSA/0.15% glycine for 15 min. For BrdU labeling, after permeabilization, cells were treated with 2 M HCl for 20 min and incubated in a borate buffer (pH = 9) for 5 min at room temperature. For the CtIP labeling, after PFA, cells were additionally incubated in ice-cold methanol and acetone for 30 min and 1 min, respectively. Then, a blocking step was performed in 1x PBS/1% FBS/5% BSA. After the blocking step, primary antibodies were incubated overnight at 4°C. Alternatively, BrdU antibody was incubated for 3 h at room temperature. All antibodies are listed in Supplementary Table 2. After three washes with 1x PBS/0.1% Tween 20 or after a 15 min wash in PBS/1% Triton-X-100 (for the BrdU immunofluorescence), the secondary antibodies listed in Supplementary Table 2 were incubated for 1 h at room temperature. Finally, all samples were washed, briefly rinsed with distilled water, progressively dehydrated in alcohol, and mounted on glass microscope slides with Vectashield mounting medium (Vector Laboratories, Inc., Burlingame, CA, United States) containing 0.25 μg/mL 4′,6-diamidino-2-phenylindole (DAPI).

Sample visualization and image acquisition were performed using an Olympus BX61 epifluorescent microscope (Olympus, Hamburg, Germany) equipped with a CV-M4 + CL camera (JAI, Großwallstadt, Germany) and CytoVision software (Applied Imaging, Newcastle, United Kingdom). Colocalization was determined visually as the coincidence in space of the protein of interest with each γH2AX focus. When indicated, image analysis and measurements were performed using ImageJ (Schindelin et al., 2012).

### Fluorescence *in situ* Hybridization

Cells were fixed in methanol:acetic acid (3:1) for 10 min, rinsed twice with 2x SSC, and dehydrated in ethanol. Additionally, for oligo-FISH, samples were incubated with formaldehyde-MgCl_2_ buffer for 10 min. Samples were denatured for 3 min at 73°C in formamide 70%/2x SSC buffer and underwent progressive dehydration. The Chr2 whole-chromosome probe (Vysis probes, Des Plaines, IL, United States) was also denatured at 73°C for 5 min prior to hybridization for 12 h at 37°C. Instead, the LacO-TxRed probe (5′-CATGTGGAATTGTGAGCGGATAACAATTTGTGG-3′) was hybridized for 1 h at room temperature. Following hybridization, slides were rinsed with 0.4x SSC/0.3% NP-40 for 2 min at 55°C and 2x SSC/0.1% NP-40 for 2 min at room temperature. Finally, samples were dehydrated and counterstained with DAPI (0.25 μg/mL). Samples were visualized using an Olympus BX61 microscope, as described in the immunofluorescence section.

### SensiTive Recognition of Individual DNA Ends

For the STRIDE assay, cells were seeded into 24 × 24-mm coverslips, and chromosome bridges were induced with the CRISPR/Cas9 system. Samples were fixed in PFA 4%, and coverslips were sent embedded in 1x PBS to intoDNA (Krakow, Poland). The dSTRIDE methodology was applied according to the protocol previously described by Kordon et al. (2019).

### Live-Cell Imaging

For time-lapse experiments, cells were seeded onto MatTek dishes (P35G-1.5-14-C, MatTek, Ashland, Massachusetts, MA, United States) and allowed to attach for 48 h. The day of the experiment, metaphase or early anaphase (EA) cells were identified using the RFP-H2B signal. Images from five z-stacks (with a 2-μm separation between planes) were acquired every 3 min for 30–60 min with a Zeiss Axio Observer Z1 inverted fluorescent microscope (Zeiss, Oberkochen, Germany) equipped with Zen blue software (Zeiss) and an AxioCam MRm camera (Zeiss). Cells were maintained under controlled conditions (5% CO_2_ and 37°C) during the experimental time course.

### Statistics

Fisher’s exact test was used to determine differences between categorical variables. For continuous variables, data normality was analyzed using the D’Agostino-Pearson omnibus K2 normality test. If the data were normally distributed, a *t*-test was used for unpaired statistical analysis; the Mann–Whitney test was applied for unpaired non-parametric analysis. Statistical analysis and graph plotting were performed using GraphPad Prism 8 (GraphPad Software, San Diego, CA, United States) and *p*-values < 0.05 were considered statistically significant.

## Results

We used three different methods to generate chromatin bridges: moderate-dose radiation exposure, inducible function of an ectopic kinetochore by expression of the LacI-GFP-CENPT chimeric protein in cellular clones stably transfected with LacO (Gascoigne et al., 2011; Supplementary Figure 1A), and CRISPR/Cas9-mediated DNA double-strand breaks on chromosome 2 (Supplementary Figure 1B) or chromosome 4 (Umbreit et al., 2020; Supplementary Figure 1C). In addition to chromosome bridges generated by the three methods described above, we examined those spontaneously formed in genomically unstable U2OS cells. While spontaneously formed bridges and those induced by radiation were randomly generated throughout the cell genome, those obtained by the CRISPR/Cas9 system in RPE1 cells were formed under exquisite spatio-temporal control and mostly involved the chromosomes against which guide RNAs were designed (Supplementary Figures 1B,C; Umbreit et al., 2020). As for the inducible kinetochore experimental model, although they theoretically should also be specific, we frequently observed the formation of non-specific bridges. In this model, the inhibition of the interaction between the LacI-GFP-CENPT chimeric protein and the ectopic LacO sequences is not complete and consequently, DNA bridges accumulate with cell passage.

### The Frequency of Chromosome Bridges Decreases as Cells Exit Mitosis and Progress to Interphase

**FIGURE 1:**
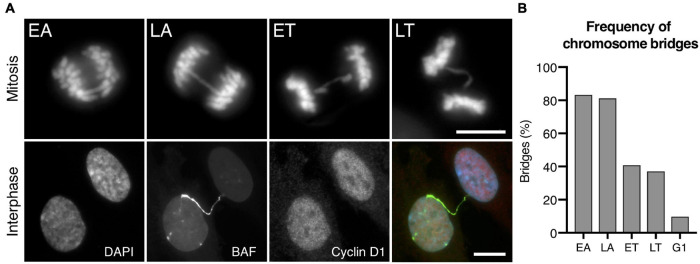
Frequencies of chromosome bridges in RPE1 Cas9 sgRNA Chr4 cells. **(A)** Representative images of bridges during the last stages of mitosis (EA, early anaphase; LA, late anaphase; ET, early telophase; LT, late telophase) and interphase. Chromosome bridges are visualized with DAPI (blue) during mitosis and with GFP-BAF (green) during interphase. To restrict the analysis to G1 cells, immunofluorescence of cyclin D1 (red) was performed, and its labeling is shown in red. Scale bar = 10 μm. **(B)** Percentage of bridges during the last stages of mitosis and in interphase cells at the G1 stage. Chromosome bridges were induced with the CRISPR/Cas9 Chr4 methodology, and cells were synchronized with RO3306 and released for 43–120 min to enrich the mitotic or interphase cell populations, respectively (*n* = 227 cells in mitosis and 1032 cells in G1).

It has been proposed that mammalian dicentric chromosomes can withstand mitotic spindle and cytokinetic ring forces to invariably persist through cell division and form long chromatin bridges between daughter cells at the interphase (Maciejowski et al., 2015; Umbreit et al., 2020). Using the CRISPR/Cas9 experimental system, we detected a progressive reduction in the frequency of chromosome bridges as cells advanced from cell division to interphase (Figure 1), a reduction that was consistent with a progressive resolution of chromosome bridges during the last stages of cell division. While we observed bridges in 83.33% of cells in EA and 81.13% in late anaphase (LA), the frequency of cells with bridges decreased to 40.91 and 37.09% in early and late telophase (ET and LT), respectively, and we only detected bridges with GFP-BAF (a sensitive reporter of chromosome bridges in interphase as its signal is not compromised by bridge stretching) in 9.79% of interphase cells at the G1 stage of the cell cycle (positive for cyclin D1). The differences in the frequencies of bridges between mitotic and interphase cells cannot be attributed to a low proliferation index as most of the interphase cells were cycling (92.12% of interphase cells incorporated BrdU after a 24-h pulse) (Supplementary Figure 2). A similar stage-associated reduction in the frequencies of bridges was also observed using other experimental systems (83.33, 58.49, 32.89, and 24.39% of irradiated MCF10A cells at EA, LA, ET, and LT, respectively, and 6.90% in interphase visualized by GFP-Lap2β; Supplementary Figure 3). These findings suggested that fundamental aspects of bridge-breakage mechanisms remained to be clarified, as the observed decrease in the frequency of bridges is not compatible with the claim that all or most bridges persist through mitosis and cytokinesis.

### Breakage of Chromosome Bridges During Mitosis

**FIGURE 2:**
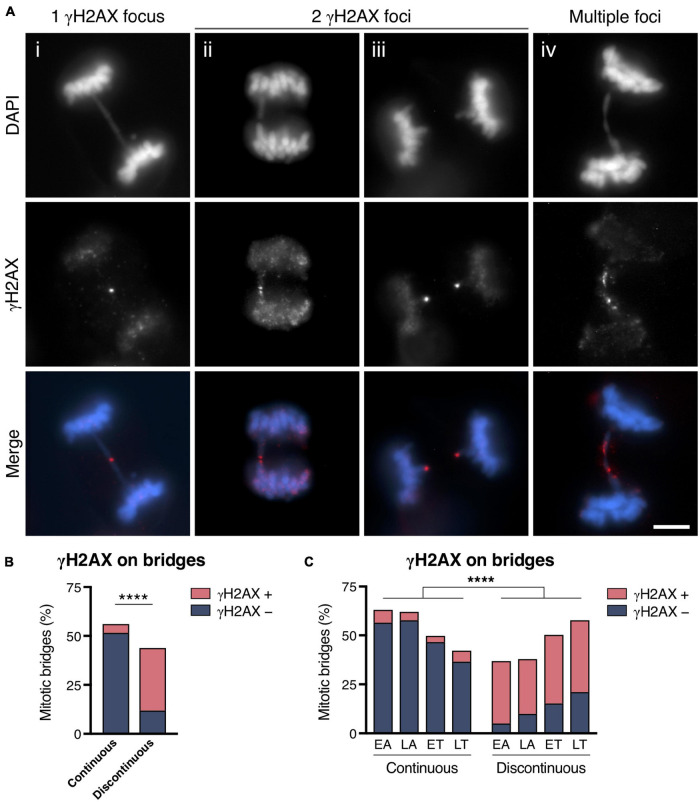
Chromosome bridge breakage is marked with γH2AX. **(A)** Representative images of chromosome bridges (DAPI, blue) that exhibit γH2AX labeling (red). Representative bridges with (i) a single γH2AX focus in the middle of the bridge, (ii, iii) two γH2AX foci flanking the discontinuity of a chromosome bridge, and (iv) γH2AX labeling spanning the chromosome bridge. Scale bar = 5 μm. **(B,C)** Frequency of chromosome bridges γH2AX-positive and -negative classified according the continuity or discontinuity of the DAPI staining for **(B)** all mitotic stages together and for **(C)** cells segregated by phase (EA, early anaphase; LA, late anaphase; ET, early telophase; LT, late telophase). Asterisks indicate statistical differences between continuous and discontinuous bridges regarding the γH2AX labeling (Fisher’s exact test, ****p < 0.0001; *n* = 609 from 5 replicates). Chromosome bridges were induced with the CRISPR/Cas9 Chr4 methodology, and cells were synchronized with RO3306 and released for 43 or 50 min to enrich the number of cells in the anaphase and telophase stages of mitosis, respectively.

Indirect evidence for mitotic bridge breakage came from the observation that bridges in mitotic cells were frequently labeled with markers of DNA rupture. To determine the extent to which mitotic bridges were labeled in a manner compatible with their severing, we immunodetected the phosphorylated form of the histone variant H2AX (then termed γH2AX), which is a chromatin marker that flags regions in the genome that contain DNA breaks (Rogakou et al., 1998, 1999). In the CRISPR/Cas9 experimental cell model, a visible discontinuity in the chromatin fiber was observed in 43.84% of mitotic bridges (considering all stages of mitosis together), and 73.03% of them presented γH2AX signaling (Figures 2A,B). Conversely, 91.52% of the continuous bridges did not show γH2AX signaling (Fisher’s exact test, *p* < 0.0001; Figure 2B). Regarding the location of the γH2AX signal on the DNA bridge (Figure 2A), three main categories were defined: (a) a single γH2AX focus usually in the middle of the bridge; (b) two γH2AX foci flanking the discontinuity of the intervening chromatin fiber; or (c) multiple γH2AX foci spanning the chromatin bridge. Interestingly, most of the continuous bridges positive for γH2AX staining displayed a single or two very close γH2AX foci in the middle of the bridge (82.14%), probably indicating a recent breakage. In contrast, the predominant labeling pattern in the discontinuous subset of chromatin bridges was a γH2AX focus located at each end of the discontinuity (76.92%). Only a small fraction (9.74%) of the discontinuous bridges positive for γH2AX displayed multiple γH2AX foci over the chromatin.

The observed association between the morphology of the bridges and their γH2AX labeling pattern was especially informative when we classified the bridges according to the stage of mitosis (Figure 2C). Whereas the fraction of continuous bridges decreased from EA (63.04%) to LT (42.25%), and most of them were devoid of γH2AX foci, bridges with visible discontinuity increased complementarily, and most of them showed a γH2AX signal. Therefore, the γH2AX-based method for the indirect detection of DNA breaks indicates that a substantial fraction of chromosome bridges are broken during the final stages of cell division.

**FIGURE 3:**
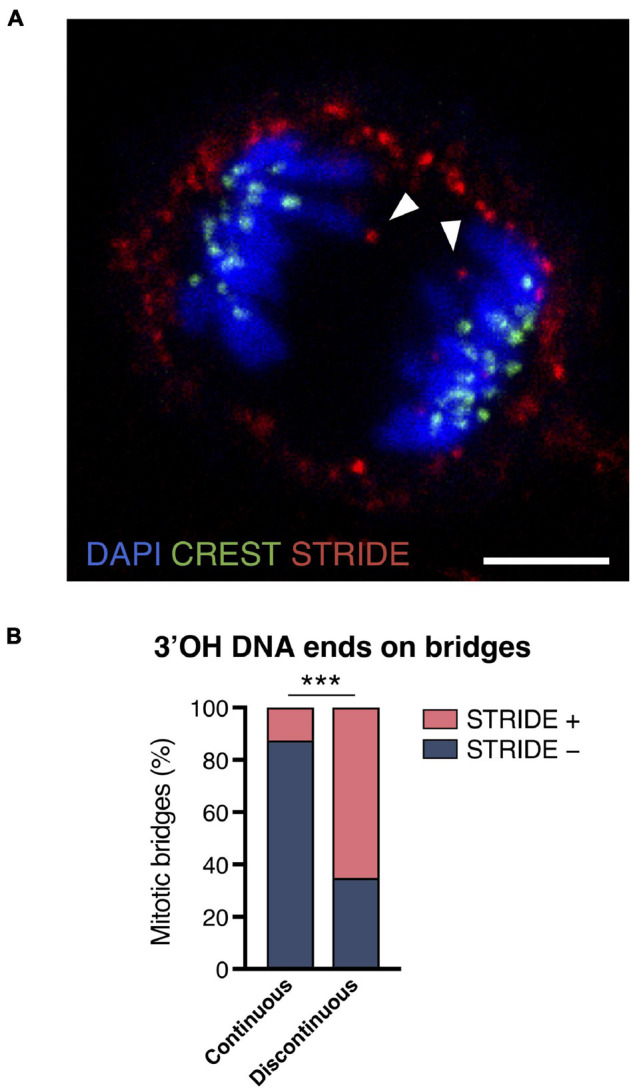
Broken chromosome bridges exhibit 3′OH DNA ends. **(A)** Representative image of STRIDE (red) detecting the DNA ends flanking the discontinuity of the chromosome bridges (DAPI, blue). Kinetochores are labeled with CREST antibody (green). Arrowheads indicate the 3′OH DNA ends of a broken bridge. Scale bar = 5 μm. **(B)** Frequency of STRIDE-positive and -negative chromosome bridges classified according to the continuity or discontinuity of DAPI staining. Chromosome bridges were induced with the CRISPR/Cas9 methodology, and cells were synchronized with RO3306 and released for 43 min to enrich the anaphase and telophase populations (Fisher’s exact test, ***p < 0.001; *n* = 82 chromosome bridges).

In addition to methods based on monitoring of histone modification at the damage sites, we used a method for the direct detection of DNA DSBs (double-strand breaks) in chromosome bridges during mitosis. The method we used is abbreviated STRIDE (section “Sensitive Recognition of Individual DNA Ends), and it enables the direct *in situ* detection of 3′OH DNA ends even when they are individual DNA double-strand cuts (Kordon et al., 2019). It consists of the conjugation of deoxynucleotide analogues to free DNA ends and their detection by hybridization with fluorescent nucleotides after rolling circle amplification. The STRIDE assay was applied to the detection of free DNA ends in compacted mitotic chromatin in RPE1 cells. In the RPE1 CRISPR/Cas9 experimental model, a total of 82 chromosome bridges were identified in mitotic cells, and 65.15% presented STRIDE labeling at the chromatin flanking the discontinuity (Figure 3). In addition, 12.5% of the continuous bridges also presented STRIDE labeling. Taken together, these results indicate that most of the discontinuities observed in chromosome bridges reflect an actual breakdown of the chromatin fiber.

### DNA Ends in Mitotic Chromosome Bridges Are Sensed as Broken Ends

**FIGURE 4:**
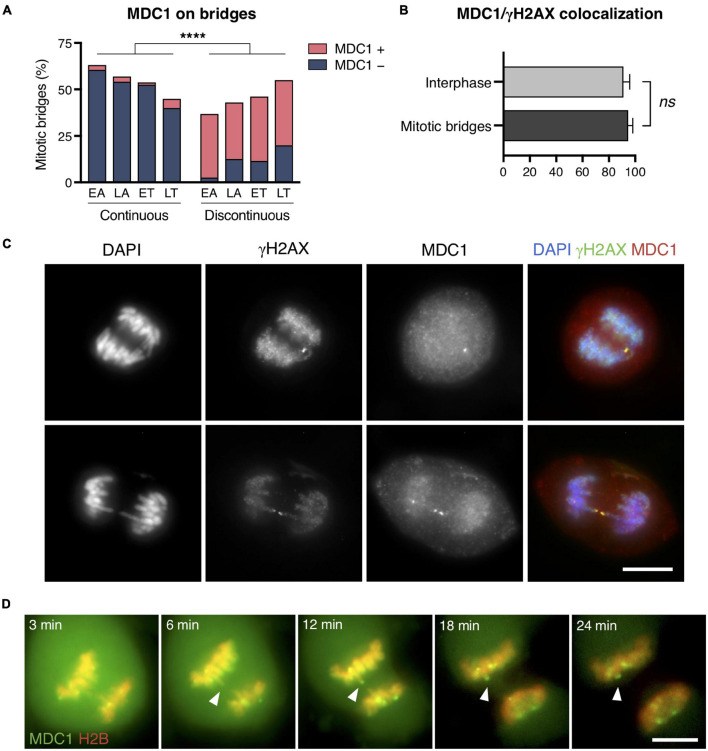
MDC1 recruitment to chromatin bridges in mitotic cells. **(A)** Frequency of MDC1-positive and -negative staining chromosome bridges during the last stages of mitosis (EA, early anaphase; LA, late anaphase; ET, early telophase; LT, late telophase). Frequencies of MDC1 recruitment to continuous and discontinuous chromosome bridges (continuity was assessed with DAPI; Fisher’s exact test, ****p < 0.0001; *n* = 208 chromosome bridges from two replicates). Chromosome bridges were induced with the CRISPR/Cas9 Chr4 methodology, and cells were synchronized with RO3306 and released for 43 or 50 min. **(B)** Percentages of MDC1/γH2AX colocalization in interphase cells *vs.* in chromosome bridges during mitosis. Error bars indicate SD (Fisher’s exact test, *ns p* > 0.05; *n* = 213 γH2AX foci in interphase cells, *n* = 146 γH2AX foci in mitotic bridges; two replicates). **(C)** Representative images for the colocalization of MDC1 (red) and γH2AX (green) at chromosome bridges (DNA in blue). Scale bar = 10 μm. **(D)** Representative images of a time-lapse of MDC1 (green) recruitment to chromosome bridges (red) in U2OS GFP-MDC1/RFP-H2B cells. Images from anaphase entrance to 24 min later are shown. Bridges were spontaneously induced. Arrowheads highlight an MDC1 focus at the end of a broken bridge. Scale bar = 10 μm.

Upon appearance of a DSB, cells activate the DNA damage response (DDR), which comprises two main stages: the initial detection of DNA breaks followed by downstream events leading to cell cycle arrest and DNA repair (Jackson and Bartek, 2009). Numerous factors involved in DNA break signaling, processing, and repair accumulate at damaged sites and form focal structures. However, although the recruitment of DDR factors to DSBs in interphase cells is efficient, the condensed structure of chromatin in mitosis is known to make it difficult to recruit or retain DDR factors to the chromatin flanking the broken ends (Giunta et al., 2010; Heijink et al., 2013; Alcaraz Silva et al., 2014). Given the important role of MDC1 (mediator of DNA damage checkpoint 1) in the early steps of the DDR (Coster and Goldberg, 2010; Jungmichel and Stucki, 2010), we investigated its recruitment to broken bridges in the CRISPR-cell model by immunodetecting MDC1 in fixed cells. We found that, similar to γH2AX, MDC1 labeled the chromatin flanking chromosome bridge discontinuities (Fisher’s exact test for MDC1 labeling continuous and discontinuous bridges, *p* < 0.0001; Figure 4A). Furthermore, given that γH2AX provides a docking site for the DDR-mediator protein MDC1, we wanted to assess whether γH2AX and MDC1 coincided at the broken ends of chromosome bridges. We found that MDC1 colocalized with γH2AX in the bridges of mitotic cells as it does in interphase cells (94.52 and 91.08% colocalization in mitotic bridges and interphase cells, respectively; Fisher’s exact test, *p* = 0.31; Figures 4B,C). In a complementary manner, the MDC1 signal was absent from those bridges without γH2AX labeling.

Next, we evaluated in living cells the dynamics of MDC1 recruitment to chromatin bridges in mitosis. To this end, we used a human osteosarcoma cell line U2OS stably expressing GFP-tagged MDC1 and RFP-H2B in which the endogenous MDC1 was knocked out using CRISPR/Cas9 (Leimbacher et al., 2019). Time-lapse recording of living cells showed recruitment of the fluorescent protein on bridges in mitotic cells followed by retraction of the chromatin fiber (Figure 4D and Supplementary Video 1). Therefore, we conclude that DNA ends in broken bridges of mitotic cells are detected and signaled by the DDR cellular machinery.

**FIGURE 5:**
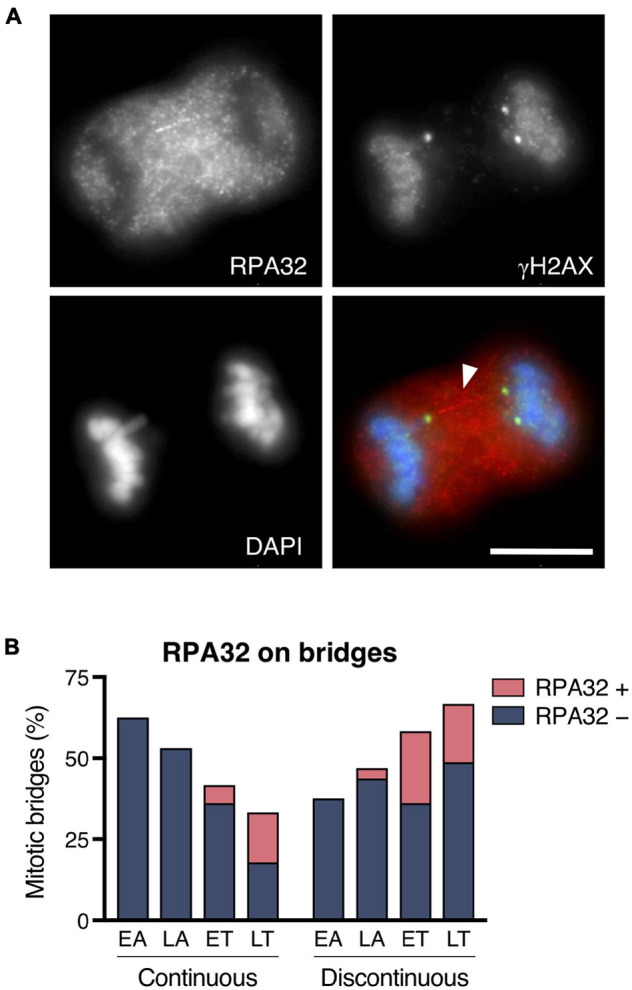
Replication protein A recruitment to chromosome bridges in mitotic cells. **(A)** Representative image for the RPA32 labeling on chromosome bridges. RPA32 (red), flanked by γH2AX (green), forms a filament-like structure that connects the two groups of segregated chromosomes (blue). Arrowhead highlights the RPA filament. Scale bar = 10 μm. **(B)** Distribution of RPA-positive and -negative staining in continuous and discontinuous chromosome bridges during the last stages of mitosis (EA, early anaphase; LA, late anaphase; ET, early telophase; LT, late telophase). Chromosome bridges were induced with the CRISPR/Cas9 Chr4 methodology, and cells were synchronized with RO3306 and released for 43 or 50 min (*n* = 127 chromosome bridges).

When instead of DSBs the lesion produces single-stranded DNA fragments, replication protein A (RPA) is known to detect them and act as a platform to recruit other factors involved in DNA repair. Using an anti-RPA32 antibody in the CRISPR/Cas9 Chr4 experimental model, we found that although most mitotic bridges showed no RPA32 signal, in 22.05% of the bridges in mitotic cells, we observed a long RPA32 signal extending like a filament physically connecting the two groups of recently segregated chromosomes (Figure 5A). As shown in Figure 5B, RPA32 signaling was mostly detected at telophase, indicating that it might require some time for RPA32 to be visible after DNA breakage. When present, the RPA32 signal coincided with the chromatin discontinuity, and it was frequently flanked by γH2AX signals (41.30%; *n* = 46 chromosome bridges). Therefore, our results indicate that bridge rupture in mitosis does not always proceed through DSB formation. Sometimes, nicks can occur that affect only one strand of DNA with the subsequent formation of long RPA32-labeled single-stranded DNA fragments. Altogether, we conclude that chromosome bridges severed during mitosis exhibit apical aspects of the DDR, as they are marked with γH2AX and MDC1 and sometimes with RPA.

### DNA Ends in Mitotic Chromatin Bridges Are Not Processed for Repair

**FIGURE 6:**
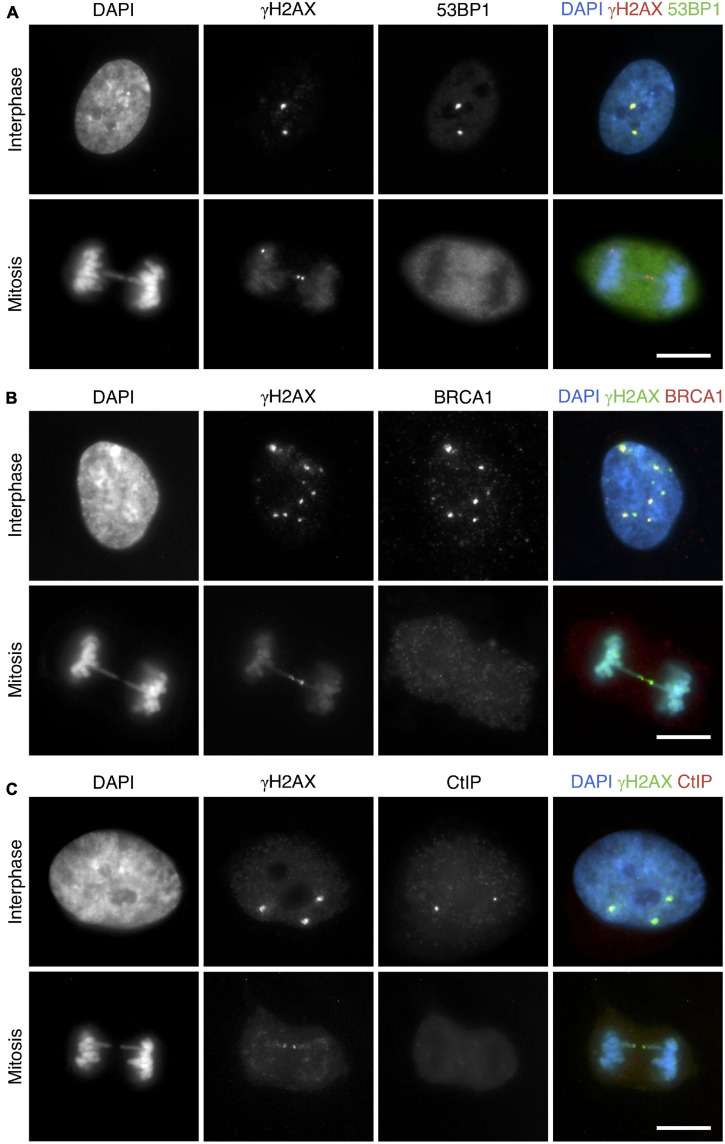
53BP1, BRCA1, and CtIP are not recruited to broken chromosome bridges in mitotic cells. Representative images for the colocalization of **(A)** 53BP1 (green) with γH2AX (red), **(B)** BRCA1 (red) with γH2AX (green), and **(C)** CtIP (red) with γH2AX (green). Upper panels correspond to interphase cells, and lower panels show mitotic cells with chromosome bridges. DNA is counterstained with DAPI (blue). All images correspond to RPE1 cells with CRISPR/Cas9-induced chromosome bridges using sgRNA Chr4. Cells were synchronized with RO3306 and fixed after a 45-min release. Scale bar = 10 μm.

Having established that mitotic cells detect and signal the broken ends of DNA bridges, we next examined the behavior of 53BP1 (p53-binding protein 1), BRCA1 (breast cancer type 1 susceptibility protein), and CtIP (CtBP-interacting protein), three proteins involved in the second stage of the DDR. At a molecular level, the balance between 53BP1 and BRCA1 plays an important role in the choice of a DSB repair pathway, and CtIP is responsible for DNA end-resection initiation (Sartori et al., 2007; Escribano-Díaz et al., 2013; Ceccaldi et al., 2016). By using an anti-53BP1 antibody in the CRISPR/Cas9 Chr4 experimental system and in irradiated MCF10A cells, we found that, in marked contrast to interphase cells, 53BP1 was excluded from mitotic chromatin (average number of 53BP1 foci per cell was 2.43 and 2.56 in RPE1 and MCF10A interphase cells, respectively, and 0 in mitotic cells). It is important to note that, whereas in interphase cells most 53BP1 foci colocalized with γH2AX foci (RPE1: 90.33%, 243/269 foci; MCF10A: 94.55%, 191/202 foci; Figure 6A upper panel), in broken mitotic bridges γH2AX did not recruit 53BP1 (RPE1: 0/71 foci; MCF10A: 0/47 foci; Figure 6A lower panel). A similar pattern was found when we detected BRCA1 (Figure 6B). Whereas focal structures of BRCA1 on γH2AX-decorated chromatin were observed in interphase cells (RPE1: 69.40% colocalization in BRCA1 positive cells in interphase, 195/281 foci; MCF10A: 56.50%, 113/200 foci), colocalization of this factor with γH2AX was absent in chromatin bridges of mitotic cells (RPE1: 0/71 foci; MCF10A: 0/53 foci). CtIP was also missing in those broken chromosome bridges marked with γH2AX (RPE1: 0/66 foci; MCF10A: 0/41 foci; Figure 6C), thus indicating that DNA end-resection was not triggered at the mitotic DNA ends. Collectively, these findings suggest that the DNA repair machinery cannot be fully launched in those bridges broken during mitosis. Thus, bridges may remain unrepaired while the ends of the broken chromosome are pulled apart.

### Breakage of Bridges in Mitotic Cells Is Associated With their Stretching

**FIGURE 7:**
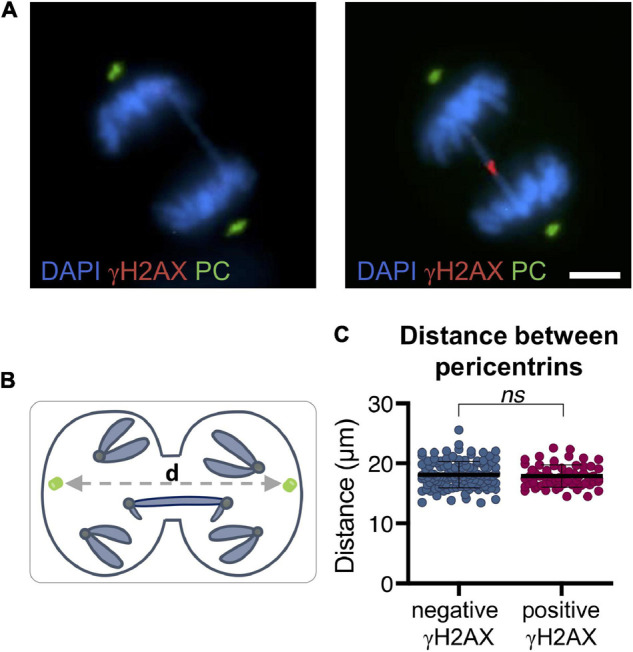
Distance between pericentrins is not associated with bridge breakage in RPE1 Cas9 sgRNA Chr4 cells. **(A)** Representative images of mitotic chromosome bridges (DAPI, blue) immunolabeled with γH2AX (red) and pericentrin (green). Scale bar = 5 μm. **(B)** Schematic illustration of the measurement of the distance between pericentrins. Pericentrins are depicted in green, *d* = distance between pericentrins. **(C)** Distance between pericentrins classified by γH2AX labeling of the bridge. The mean and SD are indicated (*t*-test, *ns p* > 0.05; *n* = 183). After CRISPR/Cas9 chromosome bridge induction, cells were synchronized with RO3306 and fixed after a 43-min release.

**FIGURE 8:**
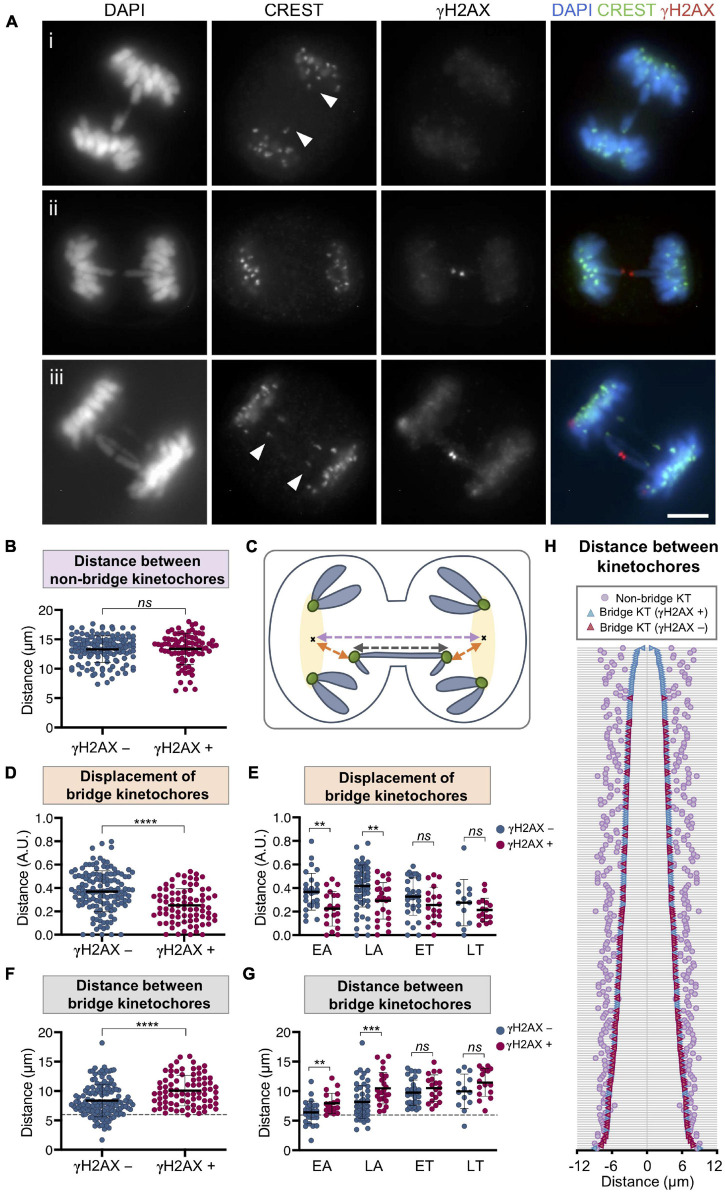
Chromosome bridge breakage is associated with the separation between bridge kinetochores in RPE1 Cas9 sgRNA Chr4 cells. **(A)** Representative images for the immunofluorescent labeling of kinetochores (CREST, green) and γH2AX (red) on chromosome bridges (DAPI, blue). (i) Arrowheads indicate the CREST signals of a continuous bridge (γH2AX-negative) that are displaced away from the non-bridge kinetochores of the cell. (ii) Representative image of a broken bridge (γH2AX-positive) with no displacement of the bridge kinetochores. (iii) Ring chromosome with one broken and one unbroken bridge sharing kinetochores (arrowheads). Scale bar = 5 μm. **(B)** Distance between the non-bridge kinetochores of cells with bridges classified according to their γH2AX labeling in unbroken (γH2AX-negative) and broken (γH2AX-positive) bridges. The mean and SD are indicated (Mann–Whitney test, *ns p* > 0.05; *n* = 216 cells from two replicates). **(C)** Schematic illustration for measurements taken of the distances between kinetochores (green): distance between bridge kinetochores (dark gray line), regions including the non-bridge kinetochores (yellow highlighted), centroid of the region (black cross), distance between centroids (pale purple line), distance between the bridge kinetochores and the centroid referred to as displacement (orange line). **(D,E)** Displacement of bridge kinetochores from the cluster of non-bridge kinetochores classified according to the γH2AX labeling of the bridge. For **(D)** all mitotic stages together and for **(E)** cells segregated by phase (EA, early anaphase; LA, late anaphase; ET, early telophase; LT, late telophase). Displacement is calculated as the distance between the bridge kinetochores and the centroid of non-bridge kinetochores (in orange in the scheme) and normalized to the distance between centroids of the cluster of non-bridge kinetochores (in pale purple in the scheme). The mean and SD are indicated. Asterisks indicate statistical differences between unbroken and broken bridges (Mann–Whitney test, ****p < 0.0001, **p < 0.01, *ns p* > 0.05*; n* = 212 from two replicates). **(F,G)** Distance between bridge kinetochores (dark gray line in the diagram) classified according to the γH2AX labeling of the bridge in broken (γH2AX-positive) and unbroken (γH2AX-negative) bridges for **(F)** all mitotic stages together and for **(G)** segregated phases. The mean and SD are indicated. Asterisks indicate statistical differences between γH2AX-negative and γH2AX-positive bridges (Mann–Whitney test, ****p < 0.0001, ***p < 0.001, **p < 0.01, *ns p* > 0.05*; n* = 212 from two replicates). Dashed line at 5.96 μm indicates the minimum distance from which bridges begin to break. **(H)** Graph displaying the distances between the kinetochores (KT) involved in the bridge (distances between kinetochores of γH2AX-negative bridges are represented in blue and those of γH2AX-positive bridges in red). Each horizontal line represents a cell. Cells are ordered according to the distance between bridge kinetochores. Pale purple dots represent the distance between the non-bridge kinetochores of each cell. All images and analyses correspond to chromosome bridges induced by the CRISPR/Cas9 Chr4 system and cells synchronized with RO3306 and fixed after 43 and 50 min of release (*n* = 212 from two different experiments).

During the last stages of mitosis, microtubules of the mitotic spindle stretch the bridging chromatin toward the opposite poles of the cell. To determine whether the increasing distance between the poles of the mitotic cell is associated with bridge rupture, we detected pericentrin, a component of centrosomes, in the RPE1 CRISPR/Cas9 Chr4 experimental model. We found no association between the distance between cell poles and the breakage of bridges in mitotic cells (signaled with γH2AX) (*t*-test; *p* = 0.46; Figure 7). In concordance with this result, we found no differences between cells with broken and unbroken bridges when measuring the average distance between non-bridge kinetochores by using a CREST antibody (Mann–Whitney test; *p* = 0.62; Figures 8A–C). Therefore, the separation between cell poles does not determine chromosome bridge breakage.

Although there was no association between bridge breakage and cell pole distance, we frequently observed that the bridge kinetochores were displaced from the cluster of non-bridge kinetochores (Figure 8A, panel i). When we measured the distance of this displacement (Figure 8C) we found that it was longer for unbroken chromosome bridges than for the broken ones (Mann–Whitney test, *p* < 0.0001; Figure 8D), thus indicating that unbroken bridges were under tension, which was released by breakage. In fact, the displacement was only significant on unbroken bridges of cells at the EA and LA stages of mitosis (Mann–Whitney test; *p* < 0.01; Figure 8E), which is precisely when mitotic spindle microtubules generate maximum tension on segregating chromosomes.

To confirm the role of mechanical stress in bridge resolution, we then measured the distance between the bridge kinetochores and found that there were significant differences between broken and unbroken bridges: the longer the distance between the bridge kinetochores, the higher the probability of bridge breakage (Mann–Whitney test, *p* < 0.0001; Figure 8F). It is important to note that this association was only observed in EA and LA, but not in telophase (Mann–Whitney test; *p* < 0.01; Figure 8G). Furthermore, when cells were classified according to the distance between bridge kinetochores, we found that in this experimental system (RPE1 CRISPR/Cas9 Chr4) a minimum distance of 5.96 μm between centromeres was needed for bridges to break (Figures 8F,G). Once the bridge kinetochores had exceeded this distance, we found broken bridges, but also unbroken bridges, which could indicate that in addition to tensile forces there may be other factors that influence the breakage of bridges during mitosis (Figure 8H). Similar results were observed for bridges induced in U2OS cells with the LacO system (Supplementary Figure 4) giving support to our conclusion.

In order to further corroborate this finding, we used the system RPE1 CRISPR/Cas9 with a sgRNA targeting the q-arm subtelomeric region of Chr2 as it allowed us to generate bridges with a different distance in base pairs between bridge centromeres (Supplementary Figure 1). While subtelomeric target sequences in Chr4 are at 139.4 and 50.7 Mb from the centromere, for Chr2 there is only one target sequence, and it is at 147.6 Mb from the centromere; thus, the intercentromeric distance is longer for Chr2 than for Chr4 bridges. Interestingly, we found that for bridges induced with the Chr2 CRISPR/Cas9 system, the minimum distance between bridge kinetochores from which bridges begin to break is 8.2 μm (Supplementary Figure 5), longer than the 5.96 μm obtained for bridges induced with the Chr4 RNA guides. This result reveals a relationship between the centromere distance in base pairs of the dicentric chromosome and the minimum separation in micrometers between bridge kinetochores from which bridge breakage occurs.

Finally, as the guide RNA for RPE1 Cas9 Chr4 targets a sequence located at the end of both, the p- and the q-arm (Supplementary Figure 1C), in some mitotic cells we identified bridging rings. This particular kind of bridges are formed when a break in the p-arm plus a break in the q-arm of chromosome 4 are induced and fusion between sister chromatids leads to one short and one long bridge (Figure 8A, panel iii). In these double bridges, we frequently found one broken and one unbroken bridge sharing only a pair of kinetochores, confirming that with the same kinetochore separation two bridges of different intercentromeric base pair length may respond differently to the tension exerted by the microtubules of the mitotic spindle. Although we cannot rule out the contribution of other causes to the resolution of chromatin bridges, our results suggest that the mechanical stress to which bridges are subjected may contribute to their resolution.

## Discussion

Our results show that there is no single cell cycle stage at which chromosome bridges are broken. Although we found that some bridges persisted beyond telophase and formed nucleoplasmic connections between the two daughter cell nuclei, most of the chromosome bridges broke during the anaphase and telophase stages. Thus, we conclude that chromosome bridges may break during the last stages of cell division. Our conclusion is based on several observations: firstly, on immunofluorescent analysis of mitotic cells, in which the discontinuous chromatin fiber of bridges is frequently labeled with intense γH2AX staining on both sides of the discontinuity. Secondly, on the time-lapse recording of mitotic cells expressing a fluorescent form of the DDR factor MDC1, as we observed chromosome bridge end retraction after MDC1 recruitment to DNA. Finally, confirmation of bridge breakage during mitosis was obtained by the STRIDE assay, as 3′-OH free ends were detected in the bridges of mitotic cells. However, bridge rupture in mitosis does not always proceed through DSB formation. Sometimes, nicks can occur in mitosis that affect only one strand of DNA with the subsequent formation of long RPA32-labeled single-stranded DNA fragments.

### The Ends of Broken Chromosome Bridges in Mitotic Cells Are Detected but Not Repaired

Although genome stability needs to be maintained during cycles for cells to pass on their hereditary material to the next generation, cells suppress DNA repair during mitosis as changes in chromatin structure necessary for repair would interfere with the correct segregation of chromosomes during mitosis (Blackford and Stucki, 2020). Thus, cells prioritize completion of mitosis over activation of a full DDR and repair of DNA damage. According to this notion, our data show that the recruitment to broken bridges of key downstream factors that regulate DSB repair pathway choice such as 53BP1, BRCA1 and CtIP is blocked during mitosis. However, the cellular response to DNA breaks is only partially disrupted in broken bridges during mitosis as upstream events such as H2AX phosphorylation and MDC1 recruitment still occur. A similar specialized response to DNA breaks in mitosis was previously described for DNA breaks induced by exposure to ionizing radiation (Giunta et al., 2010) or laser microirradiation (Alcaraz Silva et al., 2014). Furthermore, 53BP1 was found to be actively removed from mitotic chromatin as it dissociates from endogenously arising DNA DSBs at the G2/M boundary (FitzGerald et al., 2009; Nelson et al., 2009). Thus, the truncated nature of the DDR in mitotic cells does not depend on how DNA injury was induced or whether injury was induced before or after entering mitosis.

It has been suggested that the activation of early DDR events in mitotic cells may facilitate recognition of DNA damage and its repair during the following cell cycle (Giunta et al., 2010). Although this is plausible for breaks induced in most regions of the genome, it cannot be conceived of when the DNA ends are pulled apart, such as in the case of a broken chromosome bridge. It is unlikely that the two DNA ends derived from a broken bridge will be rejoined in the daughter cells, as repair proteins cannot overcome the tension of the mitotic spindle that pulls the two ends toward opposite poles (Doherty and Jackson, 2001; Blackford and Stucki, 2020). Therefore, once produced, the breakage of the chromosome bridge is likely permanent.

### Intense Stretching and Constriction of the Condensed Chromosome of Bridges in Dividing Cells

We found an association between the distance between bridge kinetochores and the probability of breakage of the chromosome bridge. These results indicate that the mechanical tension to which the chromosome bridge is subjected during the last stages of mitosis would contribute to its breakage. However, we cannot exclude the possibility that additional mechanisms, such as those based on biochemical digestion or mechanical compression, could contribute to the breakage of chromosome bridges.

Apart from the particular characteristics of each bridge, at least two additional components need to be considered to understand the behavior of chromosome bridges during mitosis: the complexity of mitotic chromosomal decondensation and the magnitude of the forces applied to the bridging chromosome. It could be speculated that, because the mitotic chromosome is highly condensed, it would decondense to prevent breakage when it is stretched to opposite poles. However, the mitotic chromosome must be able to withstand mechanical stress and viscous resistance as premature decondensation of chromatin would cause the sister chromatids to become entangled and missegregated. According to this notion, chromosome segregation and decondensation are tightly orchestrated with cytokinesis at the exit of mitosis (Kitagawa and Lee, 2015). As for the forces applied on the chromosome bridge, spindle microtubules can generate pushing or pulling forces by adding or losing subunits from their ends. At the single-molecule level, cytoskeletal proteins generate small forces. However, during cell division these proteins function cooperatively to generate forces in the range of nanonewtons and serve to accurately move separated chromatids over distances of micrometers (Scholey et al., 2003). Our results suggest that the force generated by hundreds to thousands of cytoskeletal force generators acting cooperatively must be able to break chromosome bridges during anaphase.

Considering that previous studies identified mechanical stress or biochemical digestion as possible causes of bridge breakage in interphase cells, a multifactorial model emerges for the breakage of chromosome bridges that, according to our results, can occur at different stages of the cell cycle and can obey different mechanisms. Therefore, we offer support for the validity of the BFB model as a mechanism capable of self-feeding to generate new changes in the genome.

## Data Availability Statement

The original contributions presented in the study are included in the article/Supplementary Material, further inquiries can be directed to the corresponding author/s.

## Author Contributions

AG and TA: conceptualization and supervision. MR-M: design of guide RNAs. MR-M and MS: methodology and results. DS: specialized technical support. TA, MR-M, and MS: formal analysis. AG: writing—original draft preparation, project administration and funding acquisition. TA, AG, and MR-M: writing—reviewing and editing. All authors have read and agreed to the published version of the manuscript.

## Conflict of Interest

The authors declare that the research was conducted in the absence of any commercial or financial relationships that could be construed as a potential conflict of interest.

## Publisher’s Note

All claims expressed in this article are solely those of the authors and do not necessarily represent those of their affiliated organizations, or those of the publisher, the editors and the reviewers. Any product that may be evaluated in this article, or claim that may be made by its manufacturer, is not guaranteed or endorsed by the publisher.

## Footnotes

1
https://media.addgene.org/data/plasmids/104/104993/104993-attachment_fbZFcCQmsWQB.docx

2
https://www.epfl.ch/labs/tronolab/wp-content/uploads/2019/06/LV_production.pdf
