# Supplementary material for: Breakage of CRISPR/Cas9-Induced Chromosome Bridges in Mitotic Cells
Source: Front Cell Dev Biol. 2021 Sep 28;9:745195. doi: 10.3389/fcell.2021.745195 (PMC8505897; doi:10.3389/fcell.2021.745195)
Supplement: Supplementary file 1 [file Data_Sheet_1.pdf]

Supplementary Material

1 Supplementary Figures, Video and Tables

1.1 Supplementary Figures

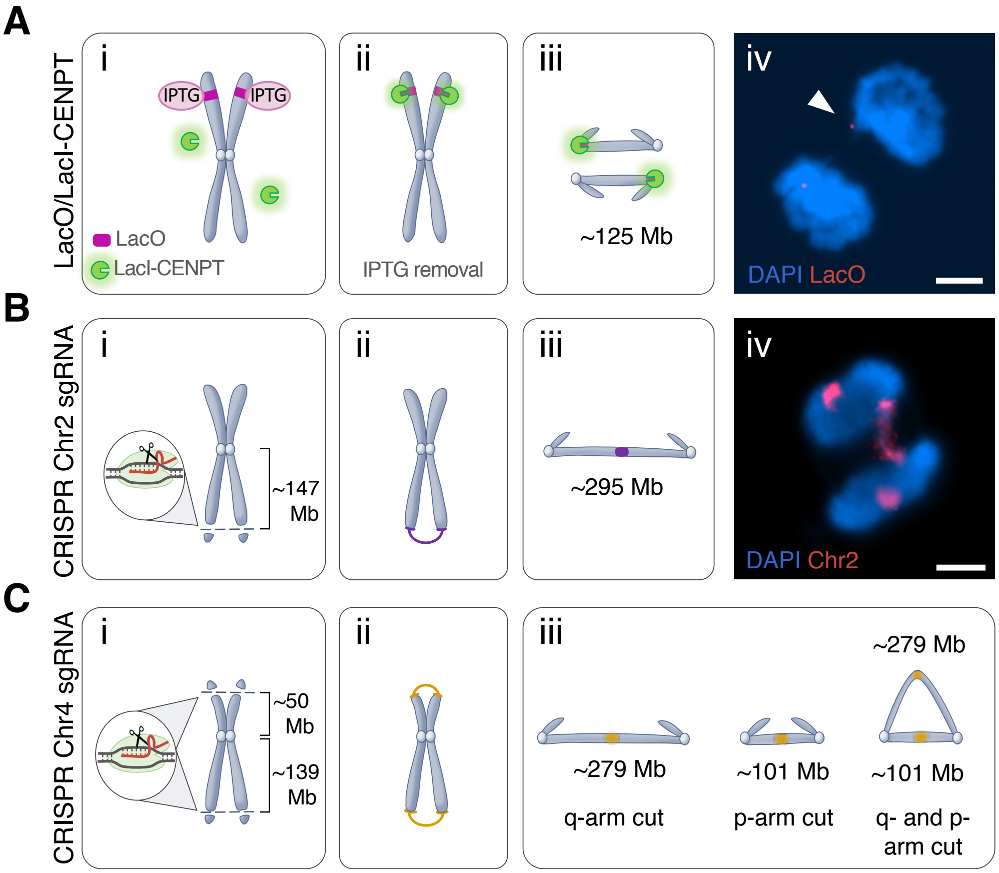

Supplementary Figure 1. Methods used for the induction of chromosome bridges of defined characteristics. (A) Schematic illustration of the LacO/LacI-GFP-CENPT experimental model to induce chromosome bridges (Gascoigne et al, 2011). (i) A LacO array (magenta) was introduced in the p-arm of Chr1. IPTG (Isopropyl β-D-1-thiogalactopyranoside) prevents the interaction between LacO and the chimeric protein LacI-GFP-CENPT (green). (ii) After IPTG removal from the media, the LacI-GFP-CENPT can interact with the LacO array and form an ectopic kinetochore. (iii) Two chromosome bridges with a distance of approximately 125 Mb between the endogenous and the ectopic kinetochores are formed during the next mitosis. (iv) Representative image of a chromosome bridge (DAPI, blue) labelled with the LacO probe (red) in U2OS cells. Arrowhead indicates the LacO signal. Scale bar = 5 µm. **(B)** Schematic illustration of CRISPR/Cas9 sgRNA Chr2 system to generate bridges. After Cas9 expression, (i) a cut in the subtelomeric region of Chr2 q-arm is produced, (ii) leading to an uncapped chromosome end, which can be repaired by fusion with the sister chromatid after replication, (iii) generating a dicentric chromosome with a distance of 295 Mb between the centromeres. (iv) Representative image of a bridge induced with CRISPR/Cas9 sgRNA Chr2 in RPE1 cells. Whole

chromosome 2 painting is shown in red, and DNA is counterstained with DAPI (blue). Scale bar = 5 μm. **(C)** Schematic illustration of CRISPR/Cas9 sgRNA Chr4 system to generate bridges (Umbreit et al, 2020). (i) sgRNA Chr4 can cut both ends of Chr4 and (ii) they can be repaired with their sister chromatid. Thus, different bridges can arise from this system. (iii) The fusion between the q-arms of chromosome 4 generates bridges of 279 Mb intercentromeric distance, and the fusion of p-arms generates a bridge of 101 Mb. When both chromosome ends are cut and fused, a ring chromosome with a long and a short arm sharing a pair of centromeres can be generated.

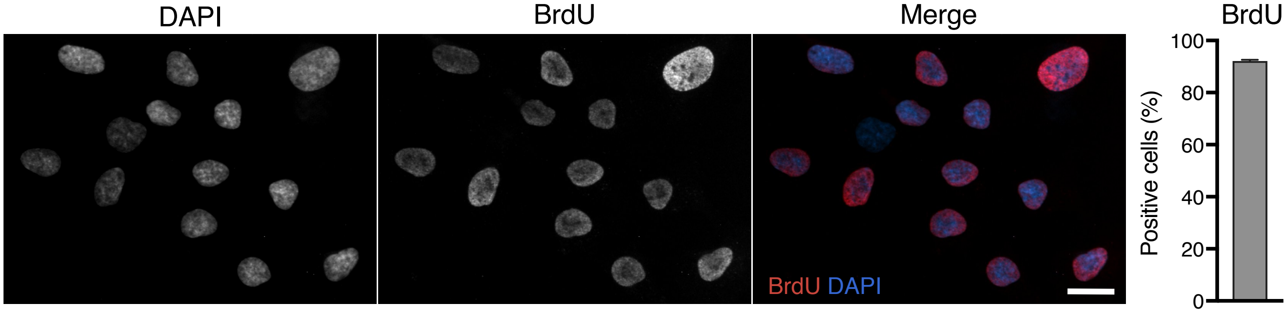

Supplementary Figure 2. Proliferation index in RPE1 Cas9 sgRNA Chr4 cells. (A) Representative image of the BrdU immunofluorescent labelling (red) after a 24-h BrdU-incubation. Cell nuclei are counterstained with DAPI (blue). Scale bar = 20 µm. **(B)** Percentage of BrdU-positive cells. Error bars indicate SD, *n* = 878 interphase cells from three independent replicates.

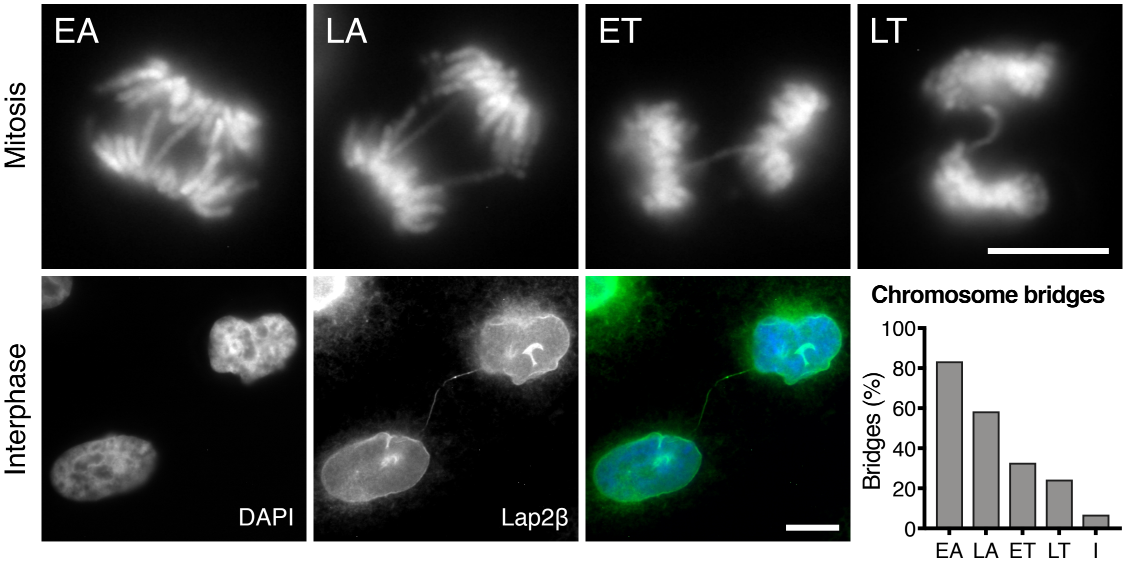

Supplementary Figure 3. Frequencies of chromosome bridges in irradiated MCF10A cells. Representative images of chromosome bridges during the last stages of mitosis and in interphase. Chromosome bridges are visualized with DAPI (blue) during mitosis and with GFP-Lap2β (green) during interphase. Scale bar = 10 µm. Graph showing the percentatge of bridges during the last stages of mitosis and in interphase (*n* = 247 for mitosis and 1493 for interphase cells). EA = early anaphase, LA = late anaphase, ET = early telophase, LT = late telophase, I = interphase.

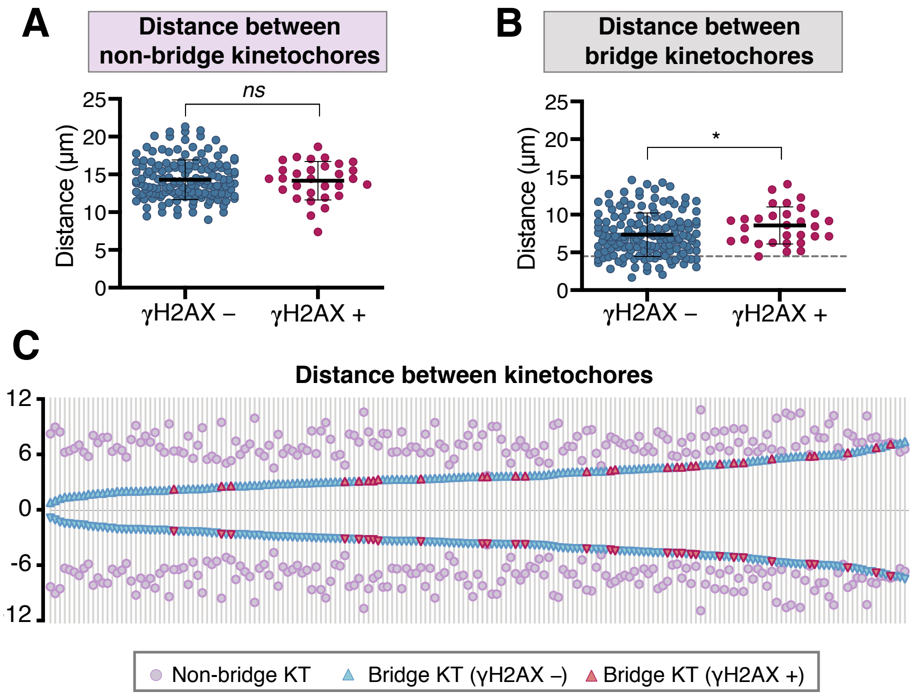

Supplementary Figure 4. Chromosome bridge breakage is associated with the separation between bridge kinetochores in U2OS LacO/LacI-GFP-CENPT cells. (A) Distance between non-bridge kinetochores in cells with broken (γH2AX-positive) and unbroken (γH2AX-negative) bridges. The mean and SD are indicated (Mann–Whitney test, *ns* $p > 0.05$; $n = 181$). **(B)** Distance between bridge kinetochores classified according to the γH2AX labelling of the bridge considering all mitotic stages together. The mean and SD are indicated. Asterisks indicate statistical differences between γH2AX-negative and γH2AX-positive bridges (Mann–Whitney test, * $p < 0.05$). Dashed line at 4.48 µm indicates the minimum distance from which bridges begin to break. **(C)** Graph displaying the distances between the bridge kinetochores (KT) of γH2AX-negative (blue) or γH2AX-positive (red) bridges. Each line represents a cell. Cells are ordered according to the distance between bridge kinetochores. Pale purple dots represent the distance between the non-bridge kinetochores of each cell. All cells were synchronized with RO3306 and fixed after a 120-minute release.

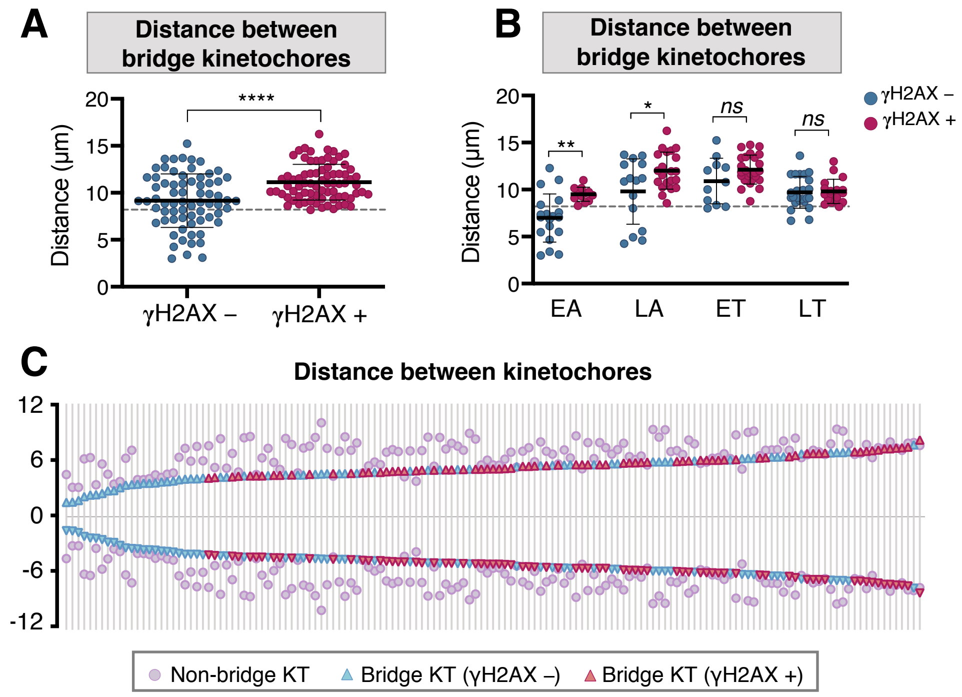

Supplementary Figure 5. Chromosome bridge breakage associated with bridge kinetochore distances in RPE1 Cas9 sgRNA Chr2 cells. (A, B) Distance between bridge kinetochores classified according to the γH2AX labelling of the bridge for **(A)** all mitotic stages together or **(B)** segregated by phase (EA = early anaphase, LA = late anaphase, ET = early telophase and LT = late telophase). The mean and SD are indicated. Asterisks indicate statistical differences between γH2AX-negative and γH2AX-positive bridges (t-test, **** $p < 0.0001$, ** $p < 0.01$, * $p < 0.05$, *ns* $p > 0.05$; $n = 145$). Dashed line at 8.2 µm indicates the minimum distance from which bridges begin to break. **(C)** Graph displaying the distances between the bridge kinetochores of γH2AX-negative (blue) or γH2AX-positive (red) bridges. Each line represents a cell. Cells are ordered according to the distance between bridge kinetochores. Pale purple dots represent the distance between the non-bridge kinetochores of each cell. All cells were synchronized with RO3306 and fixed after a 43- or 65-minute release.

1.2 Supplementary Video

Supplementary Video 1. Representative video showing MDC1 recruitment to spontaneously induced chromosome bridges in U2OS GFP-MDC1/RFP-H2B cells. The arrowhead indicates the formation of an MDC1 focus at the end of a broken bridge. The time interval between frames is 3 minutes, and the total video duration is 42 minutes. Imaging was performed with a 60x oil objective. Scale bar = 5 μm.

1.3 Supplementary Tables

Supplementary Table 1. List of plasmids used.

Plasmid	Reference	Selection	Vector Type
BAF-GFP	Umbreit *et al.*, 2020	Neomycin	Lentiviral
H2B-RFP	Addgene #26001	Cell sorter	Lentiviral
LAP2β-GFP	This study	Neomycin	Retroviral
Lenti sgRNA Chr4	Umbreit *et al.*, 2020	Puromycin	Lentiviral
Lenti sgRNA Chr2	This study	Blasticidin	Lentiviral
psPAX2	Addgene #12260	-	Packaging
pMD2.G	Addgene #12259	-	Packaging

Supplementary Table 2. List of antibodies used.

Antibody	Host	Reference	Working dilution
**Primary antibodies**			
Anti-53BP1	Rabbit	Abcam, ab21083	1:2000
Anti-BRCA1	Mouse	Abcam, ab16781, clone MS13	1:500
Anti-BrdU	Mouse	Abcam, ab8152	1:50
Anti-Cyclin D1	Rabbit	Abcam, ab16663, clone SP4	1:100
Anti-CREST	Human	Antibodies Incorporated, 15-234	1:50
Anti-MDC1	Mouse	Sigma-Aldrich, M2444, clone MDC1-50	1:250
Anti-Pericentrin	Rabbit	Abcam, ab4448	1:1500
Anti-RPA32/RPA2	Mouse	Abcam, ab2175, clone 9H8	1:500
Anti-γH2AX (Ser 139)	Mouse	Millipore, 05-636, clone JBW301	1:1000
Anti-γH2AX (Ser 139)	Rabbit	Abcam, ab81299, clone EP854(2)Y	1:500
**Secondary antibodies**			
Anti-human FITC	Goat	Antibodies Incorporated, 52-241-0100	1:100
Anti-mouse Cyanine Cy™3	Goat	Jackson ImmunoResearch Inc., 115-165-146	1:500
Anti-rabbit Alexa Fluor ®488	Goat	Thermo Fisher Scientific, A-11034	1:500
Anti-rabbit Alexa Fluor ®568	Donkey	Thermo Fisher Scientific, A-10042	1:500
